# Mesothelioma of the tunica vaginalis in a patient with giant hydrocele

**DOI:** 10.1590/0100-3984.2015.0014

**Published:** 2016

**Authors:** Cecilia Santos Montón, José Federico Ojeda Esparza, André Barbosa Ventura, Manuela Martín Izquierdo, Patricia Antúnez Plaza, Manuel Herrero Polo

**Affiliations:** 1 Complejo Asistencial Universitario de Salamanca, Spain.

*Dear Editor,*

We present the case of an 82-year-old male patient who attended our hospital emergency department complaining of pain and enlargement of his left scrotal sac. The patient reported progressive scrotal enlargement evolving over more than 20 years. He did not report any exposure to asbestos. Physical examination revealed an enlarged scrotal sac with increased temperature and testicles not easily palpable. Complete blood count revealed leukocytosis with neutrophilia (11.37 × 10^3^/µL).

Testicular ultrasonography was performed, showing left hydrocele with approximately 1100 mL in volume and dense echoes inside, making it difficult to evaluate the testis. The right testis was displaced upwards, toward the inguinal canal. Given the impossibility of performing an adequate examination of the left testis, we performed a CT scan of the testicular region. The scan revealed a large hydrocele with dense contents. The left testis showed a diffuse alteration of its structure with a lobulated margin and nodular thickening of the tunica vaginalis. During his hospital stay, the patient presented with cardiovascular instability (arterial pressure of 85/53 mmHg) and was submitted to emergency left orchiectomy with surgical drainage of the abscess, causing a complicated hydrocele.

Anatomopathological analysis led to the diagnosis of malignant mesothelioma of the tunica vaginalis testis that largely infiltrated the tunica vaginalis, testicular parenchyma and the rest of paratesticular structures (epididymis and rete testis). Immunohistochemical study showed: calretinin (+), WT1 (+), CK7 (+), EMA (+) and p53 (+).

The patient is currently receiving chemotherapy as adjuvant treatment.

Mesothelioma is a rare malignant entity that develops from serous surfaces such as the pleura, pericardium, peritoneum or the tunica vaginalis^(1,2)^. There are less than 300 cases published in the literature since the entity was first described by Barbera et al. in 1957^(3)^. Asbestos exposure is considered to be the main risk factor^(4)^. Other predisposing factors have been described, such as previous trauma, herniorrhaphy, and long-standing hydrocele^(5,6)^. The presence of a painless scrotal mass associated with reactive and recurrent hydrocele - a feature observed in more prevalent and benign testicular conditions - makes it more difficult to suspect of a malignant etiology^(1,4)^.

In the present case, the mesothelioma was diagnosed after a complication of a long standing and massive hydrocele. In the preoperative diagnosis, the paratesticular masses were interpreted as a paratesticular fibrous pseudotumor. A histological study was necessary for the diagnosis of malignant mesothelioma.

At ultrasonography, a malignant mesothelioma is characterized by the presence of a simple or complex hydrocele associated with multiple extratesticular heterogeneous masses; a single mass that grows from the scrotal wall; or an irregular focal growth of the tunica vaginalis^(7,8)^.

Radical inguinal orchiectomy with en bloc resection of the tunica vaginalis is the treatment of choice for paratesticular malignant mesothelioma. Recurrence is described in approximately 60% of cases^(1,4)^.

Malignant mesothelioma of the tunica vaginalis is a rare entity. However, it should be included in the differential diagnosis of paratesticular lesions. Considering that the tumor may be indistinguishable from other entities until histological confirmation, a radical orchiectomy is required in most cases in order to prevent late diagnosis.

## Figures and Tables

**Figure 1:**
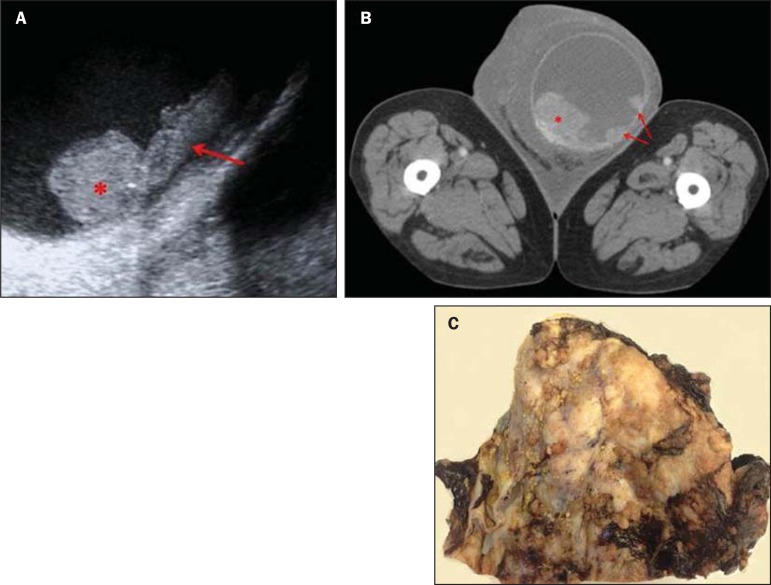
**A:** Ultrasonography of left scrotal sac: hydrocele with alteration of the testicular structure (asterisk) and nodular mass on the tunica vaginalis (arrows). **B:** Contrast-enhanced CT image of the scrotal region shows quite enlarged left scrotal sac with thickening of the scrotal envelope and presence of papillary excrescences of the tunica vaginalis (arrows). The left testicle presents a diffuse structural alteration with lobulated margins (asterisk). **C:** Macroscopic study revealed the presence of multiple papillaroid nodules in the tunica vaginalis that also showed some thickening.
